# Editing GWAS: experimental approaches to dissect and exploit disease-associated genetic variation

**DOI:** 10.1186/s13073-021-00857-3

**Published:** 2021-03-10

**Authors:** Shuquan Rao, Yao Yao, Daniel E. Bauer

**Affiliations:** 1 Division of Hematology/Oncology, Boston Children’s Hospital; Department of Pediatric Oncology, Dana-Farber Cancer Institute; Harvard Stem Cell Institute; Broad Institute; Department of Pediatrics, Harvard Medical School, Boston, MA USA; 2 School of Basic Medicine, Chengdu University of Traditional Chinese Medicine, Chengdu, China

**Keywords:** GWAS, Genome editing, CRISPR/Cas, High throughput

## Abstract

Genome-wide association studies (GWAS) have uncovered thousands of genetic variants that influence risk for human diseases and traits. Yet understanding the mechanisms by which these genetic variants, mainly noncoding, have an impact on associated diseases and traits remains a significant hurdle. In this review, we discuss emerging experimental approaches that are being applied for functional studies of causal variants and translational advances from GWAS findings to disease prevention and treatment. We highlight the use of genome editing technologies in GWAS functional studies to modify genomic sequences, with proof-of-principle examples. We discuss the challenges in interrogating causal variants, points for consideration in experimental design and interpretation of GWAS locus mechanisms, and the potential for novel therapeutic opportunities. With the accumulation of knowledge of functional genetics, therapeutic genome editing based on GWAS discoveries will become increasingly feasible.

## Background

Recent genome-wide association studies (GWAS), in which millions of genetic variants across the full allele frequency spectrum are subject to genotype-phenotype association tests, have provided insights into the genetic architecture of complex diseases over the past decades [1, 2]. As of Jan 2021, as many as 246,178 genome-wide significant associations of single-nucleotide polymorphisms (SNPs) with 868 complex traits and diseases (*P* < 5.0 × 10^−8^) have been reported (see the National Human Genome Research Institute–European Bioinformatics Institute (NHGRI-EBI) GWAS Catalog) [3]. The majority of variants found by GWAS are common variants (minor allele frequency (MAF) > 5%) in the population and have low to modest effects (OR ~ 1.05–1.20), given that current approaches for association studies are well powered to detect significant effects for such variants [4].

To obtain biological insights from GWAS requires determining the causal variants, identifying their target genes, and importantly, linking the causal variants and target genes to molecular, cellular, and physiological phenotypes [5]. Numerous strategies, including statistical methods and genomic functional annotations, have been extensively applied to prioritize causal variants (termed fine-mapping) and their target genes [6–8]; however, laboratory functional studies to validate these causal variants and their targets and to identify molecular mechanisms often lag behind. One of the ultimate goals of genetic research is to inform genomic medicine to enable more effective strategies of disease prevention and treatment. GWAS associations have led to advances in personalized medicine (i.e., individual risk prediction and optimization of therapies based on genotypes), identification of therapeutic targets, and development of novel drugs and gene therapy strategies [1, 2]. Accelerated translation of GWAS to clinical impact is highly anticipated and could alter the future of genomic medicine.

In this review, we will first discuss various experimental approaches, including classic functional experiments and more newly developed genome editing technologies, and their applications in determining the functions of GWAS-identified noncoding variants. Furthermore, we discuss both advantages and disadvantages of each experimental method, which can in turn offer guidance for study designs. We also discuss the therapeutic applications of genome editing which drive translational advances that may enable more effective disease prevention and treatment. Finally, we look ahead and discuss future challenges as our understanding of the genetic basis of complex traits evolves.

## General framework for the functional dissection of GWAS loci

**Fig. 1:**
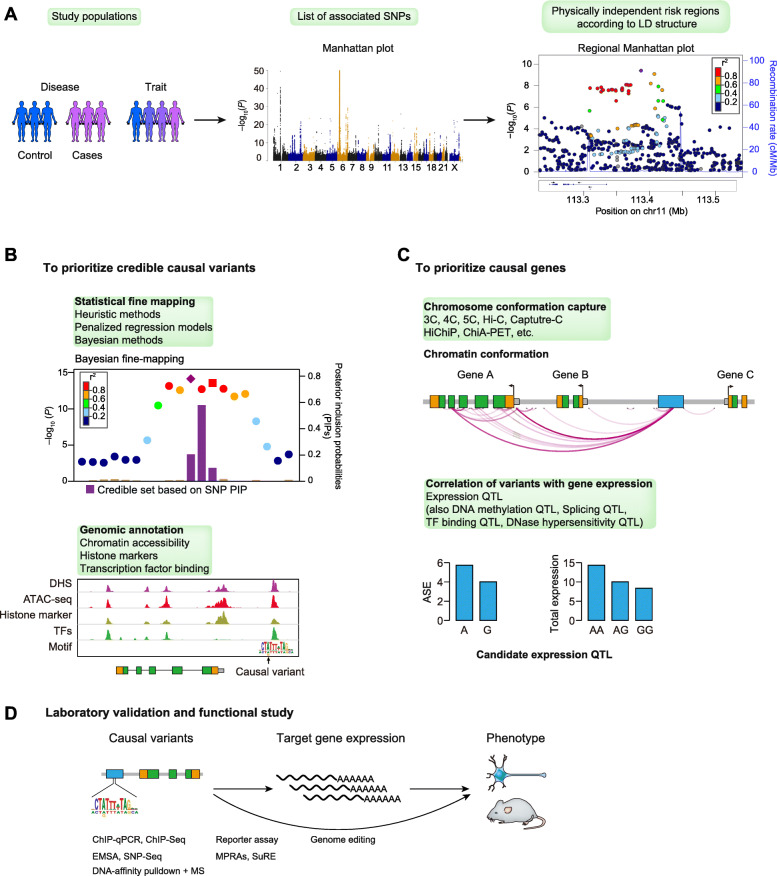
Flow of a typical process from initial GWAS to functional dissection. **a** A typical GWAS involves selection of the study populations, either case-control cohorts or general populations; genotyping of variants across the genome by single-nucleotide polymorphism (SNP) array or whole genome sequencing; and statistical analysis of variant-trait/disease associations. Regional Manhattan plots (also termed as LocusZoom plots) are generated to show the *P* values of all variants in a genomic region, to explore the patterns of linkage disequilibrium (LD) between the sentinel variant and each variant, and to annotate the genes within this region. **b** Statistical fine-mapping and genomic annotations are used to prioritize candidate causal variants. Normally, a credible set of causal variants are prioritized according to posterior inclusion probability (PIP) of each variant and genomic annotations, including chromatin accessibility, histone markers, and transcription factor binding potential, are summarized to guide the following functional studies. **c** Target genes are predicted according to enhancer-target gene promoter interaction (chromatin confirmation capture) and correlation between causal variant genotypes and target gene expression. ASE, allele-specific expression. **d** Various experimental approaches are employed to investigate the functions of causal variants and target genes and to link them back to the original phenotype

After an initial identification of variants, either common or rare (minor allele frequency (MAF) < 5%) variants, associated with a complex trait/disease, several steps may be followed for better visualization and functional dissection of GWAS associations (Fig. 1). As a first step, the GWAS list of SNPs is used to identify disease-associated region, and then each region is visualized, such as with LocusZoom plots, to identify genes within the region and the local LD structure (Fig. 1a) [9].

As multiple variants in strong LD with the causal variants tend to exhibit similar statistical significance, additional methods are required to discriminate likely functional variants from other nonfunctional correlated variants (Fig. 1b). Publicly available genomic and epigenetic annotations, such as chromatin accessibility, transcription factor (TF) binding, and epigenetic modification [10–12], may be utilized to further evaluate the functions of those selected SNPs (Fig. 1b). In addition, several computational approaches have been proposed that build upon the Bayesian frameworks for inferring regulatory hierarchies between genomic regulatory elements, which prioritize critical regulatory regions [13, 14]. One would expect that the genetic variants that fall in these elements are more likely to be disruptive for gene function. Given that the target gene(s) responsible for a GWAS signal are often not clear, identification of the causal variants could thus help determine the target genes. Statistical methods [6], genomic and epigenetic datasets, and bioinformatics tools [5, 15] used for fine-mapping have been reviewed extensively.

Normally, the target gene of a coding variant could be directly inferred according to its genomic location, and the underlying molecular mechanism could be suggested as well based on the mutation type (not further discussed in this review). However, moving from noncoding variants to target genes can be challenging given that cis-regulatory elements (CREs) may affect gene transcription over extended distances by physically interacting with their target promoters through chromatin looping interactions (Fig. 1c) [16]. Multiple lines of evidence have suggested that noncoding variants may exert regulatory effects on target gene expression (known as eQTL) or alternative splicing (known as sQTL) in trait/disease-relevant cell types [17, 18]. Thus, two categories of approaches, collectively termed regulatory target analysis, have been proposed to assign the target genes of noncoding variants: (1) eQTL or sQTL analysis to correlate variant genotypes with candidate gene expression or alternative splicing [5, 8] and (2) proximity ligation methods to delineate interactions between enhancers and target gene promoters [19]. Statistical algorithms to integrate GWAS data (both individual-level data and GWAS summary statistics) and eQTL (or sQTL) [20, 21], and technologies to investigate chromatin conformation and regulatory connections within cells, have been developed [19]. However, a recent study has revealed an inverse relationship between the proportion of *h*^2^_med_ (heritability mediated by the cis genetic component of gene expression levels) and expression *cis* heritability across genes, suggesting that genes with low expression cis heritability may have large effects on complex traits and assayed bulk eQTLs, although disease relevant, may not explain the majority of disease GWAS SNP effects [4]. Finally, laboratory functional experiments are performed in vitro in primary cell culture or in vivo in animal models, to assess the functional consequences of noncoding variants and regulatory effects on their target genes, and to investigate the mechanisms of how dysregulated genes confer risk for complex traits and disease (Fig. 1d). In the following sections, we describe experimental approaches used for functional studies of noncoding variants.

## Protein binding assays to determine the molecular functions of noncoding variants

As mentioned above, the vast majority of noncoding GWAS variants are located in CREs that are often occupied by DNA binding proteins, such as TFs [22]. To investigate the binding affinities of noncoding variants with regulatory binding proteins, several approaches have been developed, including ChIP-Seq (or ChIP-qPCR) and electrophoretic mobility shift assays (EMSAs). ChIP-Seq relies on the following hypothesis that normalized sequencing reads covering the variant are expected to be present in equal allelic ratios if the variant does not affect TFs binding, and conversely, deviations from a 50/50 allelic ratio suggest regulatory function of variants [23, 24]. Alternatively, ChIP-qPCR using allele-specific probes or primers can indicate TF binding difference to a variant between the risk and the protective allele [25]. ChIP-Seq (or ChIP-qPCR) should be performed in a cell line or tissue heterozygous for the variant of interest.

In an EMSA experiment, DNA probes surrounding a candidate variant of different alleles (~ 20–100 bp) are incubated with either purified TFs or antibodies raised against candidate TFs in vitro. Difference of electrophoretic mobility shift rate can suggest a difference of TF-variant binding affinity. Sometimes it may be difficult to predict which TFs can bind to the variant; thus, unbiased approaches such as DNA-affinity pulldown followed by mass spectrometry can be advantageous [26]. All DNA-protein complexes are first captured by a probe, and proteins specific to the risk or protective allele are then identified by mass spectrometry. To reduce non-specific binding to the DNA probe observed in conventional DNA pulldown assays, Nigrovic and co-workers developed a novel DNA pulldown method, termed Flanking Restriction Enhanced Pulldown (FREP) which leveraged distinct restriction enzyme sites on either side of the bait sequence [27]. Notably, protein-DNA interactions are regularly reported in a binary on/off manner, which is unsuitable for most noncoding variants identified by GWAS that may not act by critically disrupting the binding motif itself but instead by subtly altering the binding affinity of TFs [28]. Complete characterization of a functional noncoding variant requires knowledge not only of specificity of TF-variant interactions, but also of affinity (i.e., strength in absolute terms for a given interaction). With a semi-quantitative isobaric labeling strategy, several mass spectrometry approaches, such as thermal proteome profiling [29] and chemoproteomic approach [30], have been developed to quantify affinity of biomolecular interactions. More recently, Vermeulen and co-workers reported another quantitative binding assay which uses affinity purifications from nuclear lysates coupled with chemical labeling and mass spectrometry to quantify dissociation constants (*K*_d_^App^) of nuclear proteins for DNA and nucleosomes [31]. Protein binding assays in the context of nucleosomes may recapitulate the chromatin environment and epigenomic marks associated with a genetic variant [32].

### High-throughput protein binding assays

Protein binding assays can also be performed in high throughput which has been reviewed by Stormo et al. [33]. More recently, Li and co-workers developed an unbiased high-throughput screen, termed SNP-seq, to identify functional SNPs that allelically modulate the binding of regulatory proteins [34]. SNP-seq relies on type IIS restriction enzymes, such as BpmI, that can be directed to bind certain variants and cut at a set distance from the binding site; however, pre-binding of regulatory proteins to variants can hinder the binding of type IIS restriction enzymes, thus protecting from cleavage. By incubating a library of these variant-type IIS restriction enzyme constructs with the nuclear extract of disease-related cells or tissues, one can determine which SNPs are bound by regulatory proteins through sequencing the undigested constructs. Then, Flanking Restriction Enhanced DNA Pulldown-Mass Spectrometry (FREP-MS) can be employed to determine the binding proteins of functional variants.

### Limitations of protein binding assays

Despite extensive applications of protein binding assays in determining the function of a potential regulatory variant, they might result in false negatives when genetic variants are not supposed to disrupt a well-known TF binding motif but instead are in close proximity to the binding motifs of specific TFs [28, 35]. For instance, a major regulatory modality of red blood cell GWAS functional variants appears to affect GATA1 and co-factor binding by altering the DNA shape in the sequence-flanking core-binding motifs [35]. In addition, protein binding assays are regularly performed in vitro and may lack the appropriate biochemical context in trait/disease-relevant cell types, such as DNA and histone modifications, long-range chromatin interactions, and noncoding RNA binding. Finally, it has become clear that many of the binding sites within the genome do not affect the expression of nearby genes, serving as nonfunctional binding events [36]. These situations can produce both false-negative and false-positive results.

## Reporter assays to assess the regulatory activities of noncoding variants

Another approach complementary to the protein binding assay is the reporter assay, widely used for assessing transcriptional regulatory activity of noncoding variants [37]. When an individual variant is analyzed, the region surrounding the variant is cloned into a physiologically relevant position with respect to the reporter gene, usually luciferase or fluorescent proteins, and transiently expressed in a cell line or in a model organism. Variation in regulatory activity can then be measured by comparing reporter activity for each construct.

### High-throughput reporter assays

Instead of testing variants individually, researchers can also test tens of thousands of variants in a single experiment using massively parallel reporter assays (MPRAs) [37, 38]. For example, one study used this approach to test 32,373 variants from 3642 cis-eQTL loci for differential allelic effects and found 842 variants with differential expression between alleles, including 53 well-annotated variants linked to diseases and traits in the literature [39]. In addition to focusing on the candidate causal variants, one can use saturation mutagenesis, often by error-prone PCR [40], coupled with either the expression of a reporter gene or a sequencing-based readout to study the function of each nucleotide in a cis-regulatory element. Ahituv and co-workers performed saturation mutagenesis in conjunction with MPRA on 20 cis-regulatory elements associated with rare and common diseases, which enabled functional measurements for over 30,000 single-nucleotide substitutions and deletions [41]. Generally, saturation mutagenesis may facilitate the fine-scale evaluation of effect sizes in regulatory elements, and identification of causal variants not prioritized by fine-mapping due to unavailability of epigenetic annotations. van Steensel and co-workers further developed the survey of regulatory elements (SuRE) reporter technology with much higher throughput (> 100 fold increased scale) and resolution compared with MPRA [42]. Leveraging SuRE reporter technology, the authors survey the effect of 5.9 million SNPs, including 57% of the known common SNPs, on enhancer and promoter activity [42]. These high-throughput technologies enable rapid assessments of numerous alleles associated with a disease or trait of interest.

### Limitations of reporter assays

Several limitations and considerations should be kept in mind when interpreting the results of reporter assays. First, reporter assays typically determine the transcriptional regulatory effect of variants in a small segment of plasmid DNA, different from the native chromatin context in which the variants are located. Functional activities of enhancer candidates in a chromosomally integrated context, assessed by lentivirus-based massively parallel reporter assay, showed substantial differences from those assayed on episomes [43]. Even when reporters integrated to the genome are used, they intrinsically lack the relevant genomic context of the native variants and elements. Second, a single GWAS signal might reflect the synergistic actions of multiple co-inherited causal variants. Reporter assays are typically not designed to detect the functions of haplotypes that may include multiple variants within different regulatory elements [44, 45]. Third, both false-negative and false-positive results may occur due to the experimental design, including the size of genomic contexts and the locations of variants relative to the transcriptional start site [8]. Recent improvements in reporter assay, such as longer genomic contexts [46], different promoters or across various cell types/stages, and the ability to detect smaller effect size by the inclusion of more barcodes [47], can partially overcome these shortcomings.

## Genome editing technologies

The main limitation of the above experimental approaches is they do not test the function of variants in the native genomic context, which might therefore result in large proportion of false-positive and false-negative results. In light of these considerations, a more physiologically relevant method to investigate the functions of variants might be genome editing, which harnesses DNA repair pathways to yield desired genomic alterations within cells and organisms. Typically genome editing technologies take advantage of double-strand breaks (DSBs) introduced by programmable sequence-specific nucleases (SSNs). DNA DSBs are repaired in two ways: homology-directed repair (HDR) with a donor DNA, or non-homologous end joining (NHEJ) and microhomology-mediated end joining (MMEJ) [48]. NHEJ is the default form of DSB repair, which typically produces short insertions and deletions (indels) of a few bp in length at the cleavage site [49–51]. MMEJ, as an alternative NHEJ repair pathway that uses microhomologous sequences flanking the DSB to join the broken ends [52], is thought to be the major contributor to alleles observed after genome editing [49]. By contrast, HDR is often considered to be an error-free pathway which can use a repair template to introduce precise genetic modifications, although partial homology-driven repair events and competing NHEJ/MMEJ repair events mean that typically a range of on-target alleles are usually produced by any genome editing experiment [53]. DNA repair pathways that underlie genome editing and strategies to favor various outcomes have been reviewed extensively [54].

**Fig. 2:**
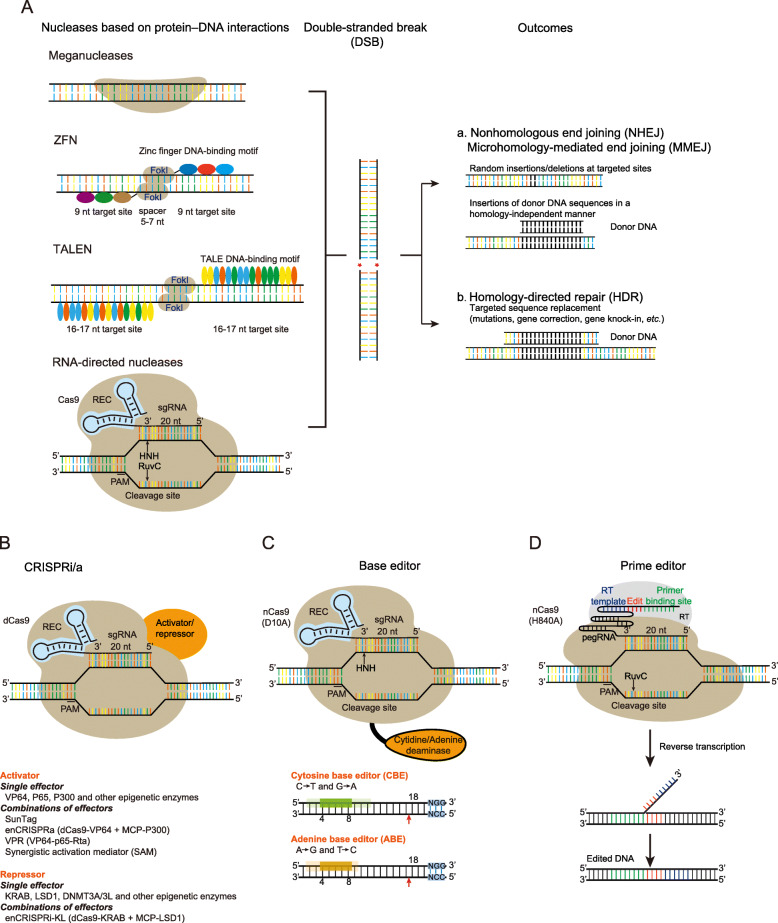
The genome editing toolbox. **A** Repair pathways of DNA double-strand breaks (DSBs) induced by nucleases. **B** CRISPR-mediated gene interference (CRISPRi) or gene activation (CRISPRa) regulates the activity of CREs by sterically blocking or changing epigenetic modifications. **C** Base editors used a fused deaminase to catalyze the conversion of C to U (C base editor) or A to I (A base editor) on one strand, followed by DNA repair on the non-edited strand. Eventually, the original C:G (or A:T) is converted to T:A (G:C) during DNA replication. **D** Primer editing leverages nCas9 to nick the genomic target, after which the reverse transcriptase copies the sequence information from the prime editing guide RNA (pegRNA) and replaces the original sequence at the target site

Tremendous effort has been dedicated to developing sequence-specific nucleases (SSNs) that are capable of efficiently introducing targeted DNA breaks [55]. To date, four different types of programmable SSNs have been developed: meganucleases, zinc-finger nucleases (ZFNs), transcription activator-like effector nucleases (TALENs), and CRISPR-associated (Cas) nucleases (Fig. 2). Despite their recent discovery, continuous improvements have made CRISPR/Cas systems a widely adopted, low-cost, easy-to-use targeted genetic manipulation tool that has been extensively applied in many organisms.

### CRISPR/Cas system

The CRISPR/Cas system, comprising CRISPR repeat-spacer arrays and Cas proteins, is an RNA-mediated adaptive immune system in bacteria and archaea [56]. According to their Cas genes and the nature of the interference complex, CRISPR/Cas systems are divided into two classes that have been further subdivided into six types and 33 subtypes [57], and new CRISPR systems are continually being discovered and repurposed. Class 1 CRISPR-Cas systems (types I, III, and IV) have effector modules composed of multiple Cas proteins that form a CRISPR RNA (crRNA)-binding complex, whereas class 2 systems (types II, V, and VI) have a single, multidomain crRNA-binding protein that is functionally analogous to the entire effector complex of class 1. The most well-characterized and widely used Cas effector is SpCas9 from the species *Streptococcus pyogenes* SF370 [58–60]. After repurposing the CRISPR/Cas9 system for gene editing, the CRISPR/Cas9 system has two components: the Cas9 nuclease and a guide RNA, either as separate crRNA and trans-activating crispr RNA (tracrRNA) components or a chimeric single-guide RNA (sgRNA) [61]. DNA binding occurs at a 20-base-pair DNA sequence (called the protospacer) that is complementary to a 20-nucleotide sequence in the guide RNA (spacer sequence) and that can be readily altered for different genome targeting [61, 62] (Fig. 2). The DNA recognition site must be adjacent to a short motif (the protospacer adjacent motif or PAM) that acts as a switch, allowing SpCas9 to bind within the target sequence [61, 62]. The requirement of PAM sequence, like 5′-NGG-3′ for SpCas9, largely restricts the genomic regions targeted by CRISPR/Cas systems. To increase the scope of targetable genomic regions, researchers have engineered Cas proteins to recognize broader PAMs by structure-guided design or directed evolution [58–60]. In addition to SpCas9, other natural CRISPR nucleases of diverse PAM sequence requirement have been also engineered for genome editing [63–66], which greatly expand the scope of target loci amenable to RNA-guided genome editing.

### CRISPR-mediated inhibition (CRISPRi) and activation (CRISPRa)

CRISPR-Cas9 has two catalytic domains (HNH and RuvC), and inactivation of both domains by point mutations (D10A and H840A for SpCas9) results in complete loss of DNA cleavage activity (catalytically inactive dead Cas9, dCas9) [67]. Without changing the DNA sequence of a given genome, fusion of dCas enzymes to effector domains enables efficient transcriptional regulation, including CRISPR-mediated inhibition (CRISPRi) and activation (CRISPRa) [67]. Notably, dCas9 itself can strongly bind to the DNA target sequence and the tight binding interferes with the accessibility of other DNA binding proteins (i.e., endogenous TFs and RNA Polymerase II) to target sequence [67, 68]. Fusing a strong repressor, such as Kruppel-associated Box (KRAB) [69] and DNMT3A/3 L [70], to dCas9, results in stronger gene repression than dCas9 alone. On the contrary, dCas9 fused with transcriptional activators can result in robust induction of target gene expression. Various activators (e.g., VP64 [71], P300 [72], ad P65 [73]), and combinations of effector proteins by dCas9 fusions and/or MS2-MCP scaffolding, including the synergistic activation mediator (SAM) system [74], SunTag [75], VP64-p65AD-Rta (VPR) [76], and enCRISPRa [77], have been developed. These technologies have largely enriched the genome editing toolbox, allowing dynamic spatial and temporal control of gene activation.

### Base editing

Base editing can generate precise point mutations in genomic DNA or in cellular RNA without generation of DSBs or a DNA donor template [78, 79]. DNA base editors are composed of a base modification enzyme (cytidine deaminase or adenine deaminase) and a catalytically impaired Cas nuclease that operates on single-stranded DNA (ssDNA) but not double-stranded DNA (dsDNA) (Fig. 2). Two types of DNA base editor have been developed: cytosine base editors (CBEs) which convert a C•G base pair into a T•A base pair, and adenine base editors (ABEs) which convert an A•T base pair into a G•C base pair. CBEs have a third fused component, uracil glycosylase inhibitor, which disfavors base excision repair and promotes mismatch repair, substantially increasing the efficiency of C•G to T•A conversion. CBEs and ABEs can collectively achieve four possible transition mutations (C to T, G to A, A to G, and T to C). Before base editing was developed, the introduction of a precise mutation usually required CRISPR/Cas-mediated HDR occurring at a DSB site in a genome via a donor DNA template harboring the desired change [80]. However, due to restriction in the G2 and S phases of cell cycles, inefficient HDR is typically observed in non-dividing cells [81, 82]. Moreover, the majority of edited products will usually contain small insertions or deletions (indels), resulting from competition between NHEJ/MMEJ and HDR [83]. In contrast, base editing does not create a DSB and therefore provides precise genome editing with a high frequency of intended as a fraction of all modified alleles. The development of various base editors and their application in sequence diversification and other areas have been reviewed extensively [84–87].

### Prime editing

Although base editing can efficiently install the four transition mutations without requiring DSBs, base editors (ABE and CBE) cannot yet efficiently perform the eight transversion mutations, as well as small insertions and deletions, although new base editors may generate C>G transitions [88, 89]. Recently, Liu and co-workers developed prime editing, a “search-and-replace” genome editing technology which can precisely install all 12 possible base-to-base conversions, small insertions, small deletions, and their combinations into target DNA sites, without requiring DSBs or donor DNA templates [90]. Prime editors contain two components: a reverse transcriptase (i.e., engineered M-MLV RT) fused to an RNA-programmable nickase (nCas9, H804A) and a prime editing guide RNA (pegRNA) that guides the prime editors to the target site and encodes the desired sequence (Fig. 2).

In comparison with base editors, prime editors induce base substitutions in more extended regions (from 3 bp upstream to 29 bp downstream of a PAM) with fewer bystander mutations at the targeted locus and at predicted off-target sites [90]. Furthermore, prime editors were used to perform insertions even up to 44 bp and deletions up to 80 bp [90]. However, there are a number of variables that need to be optimized for prime editing including the pegRNA and often a second nicking sgRNA (ngRNA) to nick the non-edited strand, which makes the experimental design more complicated than typical CRISPR gene or base editing applications [91]. The factors that affect prime editing efficiency are beginning to be clarified [92].

## Applications of genome editing technologies in functional studies of GWAS loci

For a GWAS of interest, genome editing technologies have offered a host of strategies to modify the causal variants and local CREs in physiologically relevant contexts, either in vitro in primary cell culture or in vivo in animal models, making it feasible to investigate their functions and target genes, and more importantly identify their role in determining the original phenotype. Diverse genome editing strategies across the genome editing toolbox have been conceived to modify causal variants or disrupt harboring CREs to dissect GWAS (Table 1).

**Table 1:** Summary of studies that employ genome editing technologies to investigate the functions of GWAS loci

Trait/disease	Index SNP (or causative SNP)	Coding or non-coding	Target gene	Technology	Strategy	Model	Reference (PMID)
Fetal hemogblobin	rs1427407 and rs7606173	Regulatory	*BCL11A*	TALEN	Genomic deletion	Mouse erythroleukemia cells and pre-B lymphocyte cells	24115442
Breast cancer	rs2981578	Regulatory	*FGFR2*	ZFN	Allele substitution	MCF7 cells	24265722
Hypertension	rs5603	Coding	Agtrap, Mthfr, Clcn6, Nppa, Nppb, and Plod1	ZFN	Target gene knockout	Rat	24006081
Colorectal cancer	n.a.	Regulatory	*MYC31* (possible)	CRISPR/Cas	Genomic deletion	HCT116 cells	25268989
Hypertension	n.a.	n.a.	*Nr2f2*	ZFN	Target gene knockout	Rat	25687237
Coronary artery disease	rs9349379	Regulatory	*PHACTR1*	CRISPR/Cas	Genomic deletion	Human embryonic stem cells (hESCs)	25838425
Obesity	rs1421085		*IRX3* and *IRX5*	CRISPR/Cas	Allele substitution	Human primary adipocytes	26287746
Prostate cancer	rs339331	Regulatory	*RFX6*	TALEN	Allele substitution	22Rv1 cells	26398868
Parkinson	rs356168 and rs3756054	Regulatory	*SNCA*	CRISPR/Cas	Allele substitution and genomic deletion	hPSCs	27096366
Prostate cancer	rs2742624	Regulatory	*UPK3A*	CRISPR/Cas	Genomic deletion	LNCaP cells	27409348
Type 2 diabetes	rs7903146	Regulatory	*ACSL5*	CRISPR/Cas	Genomic deletion	HCT116 cells	27539148
Colorectal cancer	rs6983267	Regulatory	n.a.	CRISPR/Cas	Genomic deletion	HCT116 cells	26743005
Ankylosing spondylitis	rs9283753		*PTGER4*	CRISPR/Cas	Allele substitution	Lymphoblastoid cell lines (LCLs)	27259153
Type 2 diabetes	N.A.	n.a.	*CDKAL1*, *KCNQ1*, and *KCNJ11*	CRISPR/Cas	Target gene knockout	hESCs	27524441
Renal cancer	rs35252396		*MYC* and *PVT1*	CRISPR/Cas	Random indels	786-O renal cancer cells	27774982
Urinary bladder cancer	rs8102137	Regulatory	*CCNE1*	CRISPR/Cas	Random indels	5637 cells	27514407
Serum acylcarnitine level	rs113569197	Coding	*SLC22A1*	CRISPR/Cas	Allele substitution	Huh7 cells	28942964
Thrombosis	rs1039084	Coding	*STXBP5*	CRISPR/Cas	Allele substitution	Mice	28062498
Schizophrenia	rs1198588	Regulatory	*MIR137*	CRISPR/Cas	Allele substitution	hiPSCs	28803920
Cardiac QT-interval	19bp indel polymorphism	Regulatory	*Rffl-lnc1*	CRISPR/Cas	Random indels and allele substitution	Rat	28827789
Vascular diseases	rs9349379	Regulatory	*EDN1*	CRISPR/Cas	Allele substitution and genomic deletion	iPSCs	28753427
Basophil production	rs78744187	Regulatory	*CEBPA*	CRISPR/Cas	Genomic deletion	HSPCs	28031487
Hypertension	rs16998073	Regulatory	*ANTXR2*	CRISPR/Cas	Target gene knockout	Rat	28077422
Blood lipid level	rs2277862, rs10889356, rs10889356 and rs10872142	Regulatory	*CPNE1* and *ERGIC3*	CRISPR/Cas	Genomic deletion, CRISPRi and allele substitution	hiPSCs, HepG2 and HEK293T cellls and Mice	28388432
Bicuspid aortic valve	rs6601627 and p.S377G (GATA4)	Regulatory and coding	*GATA4*	CRISPR/Cas	Target gene knockout	hiPSCs	28541271
Colorectal cancer	rs6983267	Regulatory	n.a.	CRISPR/Cas	Allele substitution	HCT-116 cells	29118424
Type 2 diabetes	rs780094, rs780095 and rs780096	Regulatory	*GCKR*	CRISPR/Cas	CRISPRa	HepG2 cells	28683826
Breast cancer prevention	rs9940645	Regulatory	*ZNF423*	CRISPR/Cas	Allele substitution	ZR75-1 cells and xenograft model	28821270
Red blood cell hydration and malaria susceptibility	rs10751452	Regulatory	*ATP2B4*	CRISPR/Cas	Genomic deletion	HUDEP-2 and HEK293T cells	28714864
Breast cancer and leukemia	rs11055880 and rs12142375	Regulatory	*ATF7IP*, *PDE4B*	CRISPR/Cas	CRISPRi	HEK293T cells	29061142
Prostate cancer	n.a.	Regulatory	*HOXA13*, *HOTTIP*	CRISPR/Cas	Genomic deletion	RWPE-1 cells	29117547
Pediatric chronic kidney disease	n.a.	Coding	*GREB1L*	CRISPR/Cas	Target gene knockout	Zebrafish	29100090
Height	rs9920291	Regulatory	*CHSY1*	CRISPR/Cas	Genomic deletion	T/C-28a2 cells	29205154
CKD	rs17319721	Regulatory	*SHROOM3*	CRISPR/Cas	Allele substitution	HEK293T cells	29476007
Osteoporosis	rs6426749	Regulatory	*LINC00339*	CRISPR/Cas	Genomic deletion and CRISPRi	HEK293T and hFOB1.19 cells	29706346
Prostate cancer	rs11672691	Regulatory	*PCAT19* and *CEACAM21*	CRISPR/Cas	Allele substitution and CRISPRi/a	22Rv1 cells	30033361
Osteoporosis	rs9533090	Regulatory	*RANKL*	CRISPR/Cas	Genomic deletion	U2-OS cell line	29528523
Eyebrow thickness	rs1345417 and rs12651896	Regulatory	*SOX2* and *FOXD1*	CRISPR/Cas	Allele substitution and genomic deletion	A375 cells	30248107
Idiopathic pulmonary fibrosis	rs2076295	Regulatory	*DSP*	CRISPR/Cas	CRISPRi/a	A549 cells	29924937
Prostate cancer	rs12144978 and rs4919742	Regulatory	*KCNN3* and *KRT78*	CRISPR/Cas	Genomic deletion	22Rv1 cells	30296942
Bladder cancer	rs710521	Regulatory	*ΔNTP63* and *p63*	CRISPR/Cas	Genomic deletion	5637 cells	29956121
Coronary artery disease and ischemic stroke	rs17114036	Regulatory	*PLPP3*	CRISPR/Cas	Genomic deletion and CRISPRi	HAECs cells	30429326
Primary biliary bholangitis	rs17032850 and rs227361	Regulatory	*NFKB1* and *MANBA*	CRISPR/Cas	Allele substitution	Jurkat cell lines	30528300
Hirschsprung disease, or congenital aganglionosis	p.G446R (BACE2)	Coding	*BACE2*	CRISPR/Cas	Target gene knockout and allele substitution	hiPSCs	30217742
Chronic obstructive pulmonary disease	rs2013701	Regulatory	*FAM13A*	CRISPR/Cas	Allele substitution	16HBE cells	30079747
Multiple autoimmune diseases	rs558245864	Regulatory	*BLK*	CRISPR/Cas	Random indels	LCL HG00146 cells	30478436
Biliary atresia	n.a.	n.a.	*GPC1* or *ADD3*	CRISPR/Cas	Target gene knockout	iPSCs	30358741
Pulmonary arterial hypertension	rs10958403 and rs765727	Regulatory	*SOX17*	CRISPR/Cas	CRISPRi	hPAECs cells	30527956
Alzheimer's disease	n.a.	n.a.	*FERMT2*	CRISPR/Cas	Target gene knockout	fAD and fAD^corr^ iPSCs	30371777
Colon cancer	rs6854845	Regulatory	*CXCLs (CXCL2, 3, 5, 6 and 8)*, *EREG* and *EPGN*	CRISPR/Cas	Allele substitution	HC, HCT-116 and SW-480 cells	31078271
Total cholesterol and low-density lipoprotein cholesterol	rs3780181	Regulatory	*VLDLR* and *SMARCA2*	CRISPR/Cas	Genomic deletion	HEK293T cells	30445632
coronary artery disease	rs8042271	Regulatory	*MFGE8* and *HAPLN3*	CRISPR/Cas	Genomic deletion	Cell model (HuH-7, relevant to CAD)	30861420
Bone mineral density	n.a.	Regulatory	*LHFP*	CRISPR/Cas	Target gene knockout	Mice	31042701
Osteoarthritis	rs4730222	Regulatory	*HBP1*	CRISPR/Cas	Allele substitution	Saos-2 cells	31164647
Cardiovascular disease	rs2366739 and rs1194196	Regulatory	*CD36*	CRISPR/Cas	Genomic deletion	K562 and Meg-01 cells	31344026
Chronic obstructive pulmonary disease	rs1690789	Regulatory	*TGFB2*	CRISPR/Cas	Genomic deletion	Primary human lung fibroblasts	31343404
Coronary artery disease, blood pressure, and hypertension	rs17163363	Regulatory	*AIDA*	CRISPR/Cas	Genomic deletion	TeloHAEC cells	31287004
Ventricular conduction system function	rs13165478 and rs13185595	Regulatory	*HAND1*	CRISPR/Cas	Allele substitution and genomic deletion	Mice	31366290
Neuropsychiatric disorder	n.a.	Regulatory	*CDK5RAP3*, *STRAP* and *DRD2*	CRISPR/Cas	Genomic deletion and CRISPRi	i3N iPSCs (Excitatory neurons induced from i3N iPSCs)	31367015
Primary open-angle glaucoma	n.a.	n.a.	*CAV1*	CRISPR/Cas	Target gene knockout	Trabecular meshwork cells	30916825
Type 2 diabetes	n.a.	n.a.	*ABCC5*	CRISPR/Cas	Target gene knockout	Mice	31338999
Schizophrenia	Multiple	Regulatory	*FURIN*, *SNAP91*, *TSNARE1* and *CLCN3*	CRISPR/Cas	Multiplexing, allele substitution and CRISPRi/a	iPSC	31548722
Atrial fibrillation	rs2595104	Regulatory	*PITX2*	CRISPR/Cas	Genomic deletion	Mice	31636200
Age-related hearing loss	c.539G > A, p.R180Q (SLC9A3R1)	Coding	*slc9a3r1*	CRISPR/Cas	Allele substitution	Zebrafish	30863428
Crohn's disease	rs6651252	Regulatory	*MYC*	CRISPR/Cas	Genomic deletion and CRISPRi	HCT116 and DLD-1 cells	30794691
Autoimmune diseases	rs2476601 and rs1893217	Coding and regulatory	*PTPN22, PTPN2*	CRISPR/Cas	Target gene knockout	Primary human CD4+ T cells.	31722988
Type 2 diabetes	rs534870	Regulatory	*SPRY2*	CRISPR/Cas	Target gene knockout	HepG2 cells	31664995
Pubertal timing	n.a.	n.a.	*LIN28B*	CRISPR/Cas	Target gene knockout	Zebrafish	31792362
Multiple diseases	n.a.	Regulatory	*TNFAIP3*	CRISPR/Cas	CRISPRi/a	Multiple cell lines	2144282
Colon and rectal adenocarcinoma	rs11064124	Regulatory	*CD9* and *PLEKHG6*	CRISPR/Cas	Genomic deletion	HCT116 and LoVo cells	31988071
Prostate Cancer	rs10993994	Regulatory	*MSMB* and *SNHG11*	CRISPR/Cas	Genomic deletion	LNCaP cells	32065238
Polyunsaturated fatty acid metabolism	rs953413	Regulatory	*ELOVL2*	CRISPR/Cas	Genomic deletion	HepG2 cells	31928966
Major depressive disorder	rs3101339 and rs2050033	Regulatory	*NEGR1*, *MEI1*, *NHP2L1*, *CSDC2*, and *POLR3H*	CRISPR/Cas	Genomic deletion	HEK293T cells	32214206
Breast cancer	rs1024176	Regulatory	*XCL1*	CRISPR/Cas	Genomic deletion, CRISPRi and CRISPRa	BT-474 and MDA-MB-231 cells	31904872
Nonsyndromic cleft lip with or without cleft palate	rs4791774	Regulatory	*NTN1*	CRISPR/Cas	Target gene knockout	Zebrafish	31780810
Erythroid Regeneration	rs10892563	Regulatory	*ARHGEF12*	CRISPR/Cas	Target gene knockout	Zebrafish	31467124
Psoriasis	rs10979182	Regulatory	*KLF4*	CRISPR/Cas	CRISPRa	HaCaT cells	32366252
Major depressive disorder	rs70959274	Regulatory	*LINC01360*	CRISPR/Cas	Genomic deletion	HEK293T cells	32193514

### Introduction of indels nearby causal variants

**Fig. 3:**
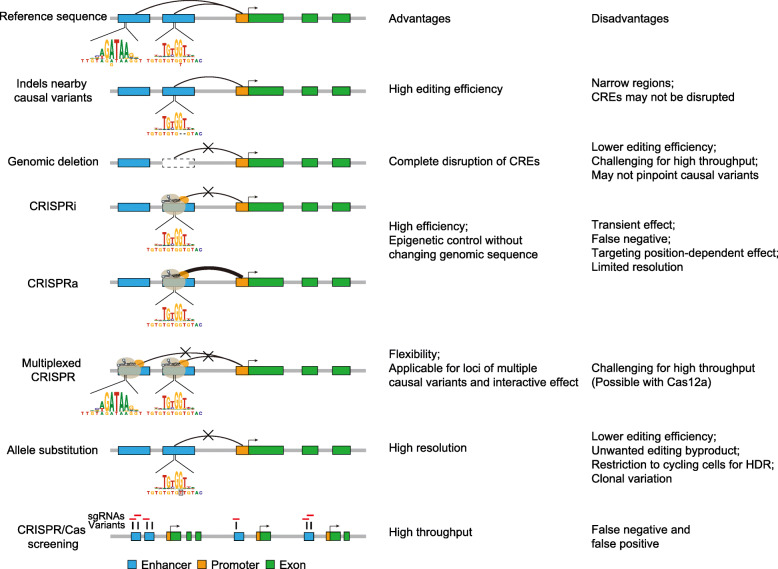
Genome editing strategies for interrogation of GWAS loci. Approaches used to investigate the functions of causal variants included introduction of indels nearby causal variants (to disrupt the core sequence of a putative transcription factor binding motif), deletion of the genomic region surrounding causal variants, CRISPRi/a (to repress or enhance the activity of local CREs), and allele substitution to change causal variants from one allele to the other (to precisely mimic the genotype). Multiplex CRISPR is used to target multiple causal variants where they can function jointly or synergistically. CRISPR/Cas screening can enable high-throughput interrogation of causal variants or target genes. Target gene knockout is typically achieved by introducing frameshifts into the coding sequence

As mentioned above, DNA DSBs can be patched through NHEJ and MMEJ repair pathways, both of which can yield varied indels without a homologous repair template [48]. The first strategy employs indels to disrupt putative CREs where the causal variants are located [49]. Given the narrow indel spectrum (a typical deletion spectrum of 1–20 base pairs) introduced by NHEJ and MMEJ, this approach requires identification of the candidate causal variants from a relatively broad GWAS risk locus (Fig. 3).

One elegant example that employed indels to disrupt causal variants is regarding an intergenic variant rs35252396, which is associated with renal cancer susceptibility [93]. Given that rs35252396 may alter the activity of hypoxia-inducible transcription factor (HIF) binding to the local enhancer, a sgRNA was designed targeting the center of the SNP-associated HIF-binding signal in 786-O renal cancer cells. After screening 36 clones of cells for indel spectra at the targeting site, the investigators identified 7 clones of cells with mutations that can affect the HIF-binding site. When compared with non-mutant clones of cells, these mutant cells exhibited significantly lower expression of target gene, *MYC* and *PVT1*. In another study, CRISPR/Cas9 genome editing was leveraged to generate two heterozygous cell lines with 6-bp or 18-bp genomic deletion surrounding rs558245864 (association with multiple autoimmune diseases) which is located in a CTCF binding motif [13]. ATAC-seq and RNA-seq in the mutant lines revealed a significant downregulation of chromatin accessibility at the focal peak and a concomitant downregulation of *BLK* expression compared with the parental cell line.

These studies suggested that introduction of indels nearby the causal variants by individual sgRNA targeting is useful for interrogation of causal variants often informed by epigenetic marks or TF binding motifs. However, small indels may be insufficient to disrupt the local TF binding motif given the flexibility of TF binding to target sites (Fig. 3), and in extreme cases, the motif will remain intact after the introduction of small indels. As a result, the consequences of indels nearby the causal variants largely depend on the degree to which local motifs are disrupted. Separation of single cell clones may be required to identify mutant cells with CREs that have disruptive deletions for downstream functional studies but this introduces the problem of clonal variation, in which numerous clones need to be studied to confidently associate gene regulation changes to gene editing, which can be especially challenging when effect sizes are modest.

### Genomic deletion surrounding causal variants

Several studies have suggested that the GWAS locus conferring risk for disease can be driven by multiple variants spanning different enhancers that target the same gene [44, 94], in which case modeling an individual variant could fail to exhibit a sufficient genetic or physiological consequence, while deletion of the entire disease risk region with multiple causal variants can be an alternative efficient strategy. Different from indels nearby causal variants, which may not disrupt the local CREs, targeted deletion of the entire genomic regions surrounding causal variants using dual sgRNAs can completely erase the local CREs (Fig. 3).

One study used CRISPR/Cas9-targeted deletion to investigate rs17114036, a common noncoding polymorphism at 1p32.2, which is associated with coronary artery disease (CAD) and ischemic stroke (IS) [95]. To determine the regulatory role of rs17114036 on target gene (*PLPP3*) expression, Yang and co-workers employed CRISPR/Cas9 to delete an ~ 66-bp genomic region enclosing rs17114036 in human aortic endothelial cells. Compared with non-edited cells, the genome-edited cells showed reduced *PLPP3* expression and altered cell behavior in agreement with PLPP3’s roles [96]. In a more recent study, Luo and co-workers identified multiple TFs binding-disruptive SNPs through integrating ChIP-Seq from human brain tissues or neuronal cells and position weight matrix (PWM) data [97]. To verify the regulatory effect of these functional SNPs on target gene expression, Luo and co-workers designed sgRNA pairs to knockout the genomic sequence containing these SNPs. Take the SNP rs3101339 for example, deletion of the genomic region (586 bp) containing rs3101339 led to significant upregulation of *NEGR1* expression [97].

Notably, the usage of dual sgRNAs can sometimes result in various outcomes, including targeted genomic deletions, inversions, and more complex genomic rearrangements, which might confound the causal interpretation of target deletion-induced phenotype in bulk cells. Single cell clones with specific editing outcomes (i.e., expected targeted deletion) may help correlate genotypes with phenotypes, although the zygosity of edits with respect to clones, particularly in aneuploid cell lines, needs to be considered carefully.

### Epigenetic control of the local CREs with causal variants

Instead of changing genomic sequences by wild type Cas9, modifying the surrounding chromatin by either CRISPRi or CRISPRa equipped by dCas9 may serve as an alternative strategy to investigate the functions of noncoding variants (Fig. 3) [67]. In addition to dCas9, fusing a catalytically inactive ZFN or TALE array to chromatin modifying enzymes can achieve epigenetic control. These non-indel forming approaches may be advantageous to achieve intermediate degrees of gene control in cases where disruption of an enhancer or other regulatory DNA may be cell lethal.

GWAS have reproducibly associated variants within intergenic regions of 1p36.12 locus with osteoporosis [98]. After prioritizing rs6426749 as a potential causal SNP at 1p36.12 through functional genomic and epigenomic analyses, two different genome editing strategies were employed to identify the potential target genes: (1) deletion of a 749-bp enhancer region containing rs6426749 using CRISPR/Cas9 and (2) epigenetically repressing the enhancer activity near rs6426749 locus using dCas9-KRAB in hFOB 1.19 cells [99]. Following both strategies, the authors observed a significant decrease of *LINC00339* expression, but not other genes nearby, suggesting that *LINC00339* is the target gene responsible for the risk locus.

Despite high efficiency for transcriptional regulation, CRISPRi and CRISPRa have intrinsic shortcomings. First, the regulatory effect by either CRISPRi or CRISPRa may be transient such that the chromatin may revert to its original state after the epigenome modifying machinery is removed, although the duration of epigenetic memory may depend on a number of variables including the specific locus and physiological context [70]. Second, the activity of CRISPRi/a is correlated with the distance of the target site from transcription start sites (TSSs) and core regulatory elements where most of the causal variants lie. For example, strong CRISPRi activity is obtained by targeting a window of DNA from 50 to + 300 bp relative to the TSS of a gene, while strong CRISPRa was observed for sgRNAs targeting 400 to 50 bp upstream from the TSS [71, 100]. For enhancer regions, strong CRISPRi or CRISPRa activity was observed when targeting DNA 100–200 bp away from the accessible chromatin regions [77]. Third, the dynamic range of gene expression control by CRISPRi/CRISPRa may vary depending on genomic and cell-type context, and may exceed or underperform the effects of actual genetic variants, suggesting the potential for false-positive or false-negative effects [101]. Finally, epigenetic modifications induced by CRISPRi repression can encompass a > 1 kb [102], thus limiting the resolution of CRISPRi for variant fine-mapping. Combinational applications of both epigenetic editing and base editing/prime editing (discussed below) might be warranted, to verify the regulatory effect of a GWAS locus on target gene expression and to pinpoint the causal variants responsible for the GWAS locus.

### Multiplexed genome editing and epigenetic control

Previous studies have suggested that complex diseases arise from the accumulation of genetic variants that are enriched in genes expressed in molecular networks [103], and individual genes must be understood in the context of molecular networks that define the disease states. More importantly, multiple causal variants may act synergistically, contributing to disease phenotype/trait variance [44, 94, 103]. Multiplexed CRISPR technologies which leverage simultaneous expression of multiple Cas proteins or gRNAs to edit or transcriptionally regulate numerous genetic loci in parallel hold promise for functional study of multiple variants from one GWAS locus (haplotype) or different loci [104] (Fig. 3). For example, in order to investigate the potential synergistic effects of schizophrenia-related genes, Brennand and co-workers used CRISPRa to upregulate *SNAP91*, *TSNARE1*, and *CLCN4* and RNA interference (RNAi) to repress *FURIN* in human induced pluripotent stem cells [105]. They observed larger effects of combinatorial perturbation converging on synaptic function, than the additive effects of individually perturbed genes. In another study, Shendure and co-workers used saturation genome editing to assess the pathogenicity of all possible single-nucleotide variants (SNVs) in 13 exons that encode functionally critical domains of *BRCA1*, a tumor suppressor gene related to both breast and ovarian cancer [106]. To introduce SNVs into haploid human cell line (HAP1), a Cas9/gRNA construct was transfected with a library of plasmids containing all SNVs within approximately 100 bp of genomic sequence (the homology arms) to favor homology-directed repair. Functional scores were systematically derived for 3893 SNVs based on cell survival, independent of prior expectation, which was immediately useful for the clinical interpretation of *BRCA1* variants.

### Allele substitution of causal variants

While it is crucial to determine the regulatory mechanisms through which a disease-associated variant could affect target gene expression, another question would be how potentially modest variation in a target gene’s expression could result in a disease phenotype, given that the majority of eQTL effects are of relatively small magnitude (< 2-fold change in expression) [107]. Despite high performance in identifying the target gene and determining the function of causal variants, either targeted deletion or epigenetic modification of a CRE may not have the same effect as that of a single-nucleotide change. Therefore, to mutate the causal variant from one allele to the other by HDR, base editing or newly developed prime editing could ensure target gene expression in a physiologically relevant manner (Fig. 3).

Recently, Jaenisch and co-workers leveraged CRISPR-mediated HDR to mutate the candidate causal variants at the *SNCA* locus, which is associated with Parkinson’s disease (PD) [108]. After prioritizing candidate causal variants based on epigenetic signatures and in silico TF motif predictions, a 500-bp genomic region containing two SNPs in human embryonic stem (ES) cells was first deleted and then the 500-bp region with either the risk or protective alleles of the two SNPs was reinserted using HDR. In comparison with cell clones harboring the protective alleles of the enhancer SNPs, clones bearing the risk-associated alleles showed significantly higher SNCA levels [108]. A more recent study focused on GWAS variants in or near the *FAM13A* (family with sequence similarity member 13A) associated with chronic obstructive pulmonary disease (COPD) [109]. Following conditional genetic association and MPRAs which together prioritized rs2013701 to be the most promising causal variant responsible for this GWAS locus, CRISPR-based homology-directed repair was applied to generate single clones homozygous for either TT or GG genotype at rs2013701 in 16HBE cell line. Compared with rs2013701 TT clones, GG clones predicted reduced expression of *FAM13A* and demonstrated an increased rate of cellular proliferation.

The current genome strategies used for allele substitution in functional studies of GWAS loci are restricted to CRISPR/Cas or other nuclease-mediated HDR. However, due to competition between the NHEJ/MMEJ and HDR repair pathway following DSBs, allele substitution using HDR is often inefficient and the occurrence of unintended indels might also cause imprecise editing of the target gene [83], thus limiting its applications in genome editing. Instead, base editing has been on its horizon.

Reiner and co-workers performed whole genome sequencing of over 62,000 ancestrally diverse participants in the TOPMed program and identified 14 single variant-red blood cell associations at 12 unique loci [110]. To further investigate the function of one sentinel variant, rs112097551, underlying red blood cell development, Reiner and co-workers used cytosine base editing to modify the reference G to alternative A allele in HUDEP-2 erythroid precursor cells. Compared to G/G clones, all five G/A heterozygous HUDEP-2 clones showed significantly reduced expression of *RUVBL1*, but not other nearby genes, suggesting rs112097551-G may exert long-range control of the gene *RUVBL1* which is essential for hematopoiesis [110]. Despite potentially higher editing efficiency and product purity, prime editing has yet been employed in GWAS functional studies.

### CRISPR/Cas screening

Analogous to the high-throughput protein binding assays and reporter assays, high-throughput CRISPR screens have been also employed in functional studies of GWAS (Fig. 3). One recent study used CRISPRi screens to dissect thousands of noncoding variants at the TNF-α-induced protein 3 (*TNFAIP3*) locus that is associated with multiple diseases [111]. Hacohen and colleagues first employed either CRISPRi or CRISPRa targeting all regions with accessible chromatin in three cell lines, to identify regions that significantly repress *TNFAIP3* expression, and then leveraged MPRAs to test for allele-specific reporter expression induced by individual variants, which finally prioritized 18 causal variants at this locus. Given the relatively broad targeting range by CRISPRi/a, identification of the causal variants required additional tools that can test individual variant, such as EMSA and reporter assay as mentioned above. Instead of CRISPRi/a, CRISPR-mediated varied indels have been also applied for screening of cis-regulatory elements [112, 113]. Previous GWAS and other human genetic studies have highlighted the association between the *HBS1L-MYB* interval and fetal hemoglobin (HbF) levels [114]. To functionally fine-map the *HBS1L-MYB* intergenic region, Canver et al. performed variant-aware saturating mutagenesis of this region in HUDEP-2 cells using multiple nucleases with different PAM sequence requirements [112]. Multiple putative functional elements were identified, including the previously known − 84 DNase I-hypersensitive site (DHS) which harbored a potential causal variant rs61028892 [112]. Again, the resolution is a function of the number of sgRNAs available within a given genomic region which in turn depends on the nuclease and its genome targeting range [60].

### Therapeutic applications of genome editing

A central objective of genetic research is to translate biological insights into clinical applications that enable effective prevention and treatment of diseases. Human disease genetics has identified thousands of mutations that result in diverse diseases, which provided insights into gene therapy strategies [1, 115, 116]. Therapeutic applications (i.e., accuracy, precision, and safety) of genome editing in monogenic diseases both ex vivo and in vivo, as well as delivery methods of genome editing tools, have been extensively reviewed elsewhere [117, 118]. Here we focused on examples of adult-onset disease to demonstrate the significant advances of translation from genetic variants identified by GWAS to disease gene therapy.

GWAS have identified multiple SNPs associated with increased expression of fetal hemoglobin and a lower severity of both transfusion-dependent β-thalassemia (TDT) and sickle cell disease (SCD) in adults [23, 119]. Some of the SNPs are located in an erythroid-specific enhancer of *BCL11A* encoding a zinc finger-containing transcription factor that represses γ-globin expression and fetal hemoglobin in erythroid cells [23]. These findings have led to a considerable effort to target *BCL11A* to increase fetal hemoglobin levels in patients with β-hemoglobin disorders [120, 121]. Disruption of GATA1 binding sequences within the erythroid-specific enhancer of *BCL11A* in hematopoietic stem and progenitor cells (HSPCs) by either CRISPR/Cas9 or base editors significantly reduced BCL11A expression in erythroid-lineage cells, restored γ-globin synthesis, and reactivated production of fetal hemoglobin, even though the core GATA1 binding sequences are not subject to common genetic variation but rather neighboring sequences [115, 116]. Both TDT and SCD patients infused with autologous CRISPR/Cas9-edited CD34^+^ HSPCs 9 targeting the GATA1 binding sequences at the erythroid-specific enhancer of *BCL11A* showed increased in fetal hemoglobin, transfusion independence, and elimination of vaso-occlusive episodes (in the SCD patients) [115]. Therefore, the therapeutic potential of editing a GWAS locus not only depends on finding the causal SNPs per se but rather in understanding the elements and genes impacted which themselves could constitute the ultimate therapeutic target. In contrast to common variants of modest effect size, low-frequency or rare variants (MAF < 5%) uncovered by GWAS, especially those leading to loss-of-function, usually exhibit a relatively large phenotypic impact [122]. For example, carriers with inactivating mutations on *PCSK9* were found to have markedly lower LDL cholesterol level and CAD risk, which led to the discovery of two FDA-approved monoclonal antibodies [123]. We argue that the interpretation of genetic variation is presently the rate-limiting step for genomic medicine. With the accumulation of functionally verified genetic variants and continuous advancement of CRISPR/Cas editing technologies, therapeutic genome editing and GWAS-inspired development of small molecules may become feasible for more and more polygenic diseases in the near future.

## General considerations for functional genetics

Although genome editing has drastically accelerated the identification of causal variants by linking these variants to target gene expression or the original phenotype, there are several general considerations that are applicable to genome editing approaches in functional studies.

### Cell type or cell state

Most of the current functional GWAS studies are performed in human cell models. As mentioned above, the vast majority of disease-associated variants reside in gene distal sequences such as enhancer elements (upstream, downstream, or in introns of target genes). Enhancers control spatiotemporal gene expression programs by engaging in physical contacts with promoters of their cognate genes, often through long-range chromosomal interactions. Since both enhancer repertoires and the enhancer-promoter interactome are highly cell type-specific [124, 125], many disease-associated variants may regulate target gene expression and cellular functions in a cell type-specific manner. Supporting this hypothesis, previous studies have revealed significant enrichment of GWAS SNPs in active regulatory regions in disease or trait-relevant cell or tissue types, compared to random SNPs [126, 127]. For a given disease or trait, several methods have recently been developed that integrate tissue-specific gene expression or genomic annotations with GWAS summary statistics to identify risk loci enrichment in specific cell types. These methods included, but not limited to, SNPsea, DEPICT, RolyPoly, g-chromVAR, and CHEERS [127–132]. Such frameworks allow researchers to narrow down potential disease-relevant cell types or states, which is crucial for designing functional follow-up experiments and gaining mechanistic insights. Notably, recapitulating the cell state(s) impacted by GWAS SNPs may include not only cell type but also environmental conditions and transient perturbations, as SNPs may only show a phenotypic difference in response to such cues.

### Cellular/physiological function

Determining the causality of a variant ideally requires demonstrating an altered phenotype following allelic replacement. Practically, this may be done in vitro in primary cell culture or in vivo in animal models. Given the potential similarity of conserved physiologic phenotypes between animal models and humans, linking GWAS variants back to the original phenotype might be more directly assessed in animal models. However, for human-specific traits, animal models may have limitations. Reciprocally, the precise functional assays that are disease- or trait-relevant can be challenging to define in human cell cultures. For example, neuronal cell types are thought to be implicated in psychiatric traits [132], but it is not known which specific neuronal functions are compromised in disease. In light of this issue, intermediate phenotypes (measurable cellular functions) might be quite useful for functional assays as long as these phenotypes are truly intermediary to the complex phenotype. A recent study showed that variants associated with susceptibility to infection tend to modulate the secretion of monocyte cytokines (cytokine QTLs) [133]. Thus, it would appear fruitful for functional validation of infection-associated variants to assess monocyte cytokine secretion.

Moreover, global changes in gene expression may be a more general and unbiased phenotype to indicate cellular functions. Recently, several technologies combining CRISPR/Cas-mediated genome editing and single cell RNA-sequencing (scRNA-Seq) have been developed, like CROP-seq [134] and Mosaic-seq [135], which enable matching the transcriptome of single cells with genetic variants introduced by genome editing. In the future, high-throughput phenotyping of human cells will be crucial for identifying the best assays to validate candidate GWAS variants or genes.

### Genome editing in primary cells

To investigate the functions of variants, researchers ideally would mimic the exact polymorphisms naturally observed in GWAS by genome editing. Although this approach can be applied in immortalized cell lines, it may be more challenging in primary cells that are not easily expanded in culture. Currently, many strategies are available to deliver Cas proteins and other editors into cells, including plasmid transfection, viral delivery, RNP, and mRNA delivery, which holds promise for high editing efficiency in primary cells [120]. For example, utilizing ts-rSeV and lentivirus to deliver SpCa9 and sgRNAs separately, we have successfully performed CRISPR/Cas9 screening in hematopoietic stem and progenitor cells (HSPCs) to study neutropenia-associated variants, leading to the identification of previously unappreciated mechanisms of neutropenia [136]. Instead of primary cells, human induced pluripotent stem cell (iPSC), which can be differentiated into diverse cell types [137], is an attractive system to study molecular consequences of genetic variants. Several studies have demonstrated that human iPSC-derived cardiomyocytes from long QT syndrome patients can faithfully recapitulate disease phenotypes, allowing scientists to study some of the disease traits in vitro [138]. Moreover, as a result of expansion potential in vitro, iPSCs allow for various types of genome editing followed by selection of clonal cell line with the accurate editing outcome. Multiple studies have integrated genome editing technologies with hiPSC-based studies to study the functions of GWAS variants and genes (Table 1).

### Animal models

Model organisms are typically preferable for experimental disease research due to similar anatomy and physiology with human beings. However, there are important differences between model organisms and human beings in terms of genetic architecture (especially noncoding genomic regions), and mutations in humans that result in specific phenotypes that may not be faithfully recapitulated in model organisms [139]. Xenograft models may serve as a compromise allowing the study of human cells in an animal setting, though these may also have some limitations in terms of how reliably the xenograft mimics the physiology of the native human tissue.

### Limitations of genome editing

Despite widespread potential applications in functional genetics, CRISPR/Cas genome editing tools still have their limitations. First, genome editing efficiency highly depends on a multitude of factors, including cell type, delivery of genome editing tools, cycling rate of cells, and mutagenesis efficiency. Second, when the CRISPR system recognizes sequences homologous to the target sequence, off-target mutations may occur, especially in mammals given their large genomes, which can lead to undesired functional impacts [140]. In addition to the production of unwanted local mutagenesis, off-target DNA cleavage can potentially give rise to chromosomal rearrangements and disrupt the functionality of otherwise normal genes and regulatory elements [141], which might impact the interpretation of the CRISPR-induced phenotype. Third, several studies have revealed that DSBs introduced by genome editing can select for inactivation of the p53 pathway, which is associated with transformation in numerous cell types [142]. Finally, there is potential for pre-existing immunity against CRISPR components to limit the feasibility and safety of in vivo delivery, given the evidence that pre-existing Cas9 antibodies and reactive T cells have been detected in humans after exposure to pathogenic bacteria of CRISPR systems [117, 143]. Such “side effects” of genome editing can typically be addressed by including appropriate empiric controls, such as performing gene editing in parallel at neutral genomic loci [144, 145] and evaluating the status of the p53 pathway.

## Conclusions and future directions

Despite thousands of SNP-trait/disease associations have been identified by GWAS, only a small fraction of them have been functionally investigated. In order to translate these associations to biological insights, one needs to determine the causal variants, target genes, and the underlying mechanisms linking variants and genes to the original phenotype. Moving beyond protein binding assays and reporter assays, which can determine the functions of variants either in vitro or in an ectopic context, genome editing technologies can manipulate variants and their harboring elements in trait-relevant chromatin, genomic, and cellular contexts. Genome editing will undoubtedly spur progress in this field and accelerate the translation of genetic advances to novel therapeutics.

In the next decade, we foresee several important areas where advances are likely to occur. First, functional experimental approaches, especially in high-throughput, performed in trait/disease-relevant cell types will be further developed. Current functional assays, such as SNP-seq [34], largely rely on cell lysates from cell lines, which may lead to both false-positive and false-negative TF-DNA binding as discussed above. These assays could benefit from using either primary cell lysates or three-dimensional organoid cultures. Second, although genome editing technology has been rapidly advanced by fine-tuning the architecture of nucleases, i.e., Cas proteins, to increase the efficiency, specificity, and targetability, there remain many challenges to be overcome before its full potential can be realized. For instance, despite the superiority of human primary cells to model trait/disease phenotype, functional studies of GWAS loci in human primary cells have been scarcely reported, possibly due to low editing efficiency. Third, in addition to single-nucleotide variants, disease-associated structural variations where differences in genomic DNA can range from kilobase to chromosomal magnitude have been discovered through whole genome sequencing. Several genome editing-assisted methods have been developed for targeted insertion, deletion, or replacement of long sequences and genes [146], providing efficient tools to study these structural variations. Finally, despite the inevitable time lag from basic research to clinical implementation, a growing number of examples have highlighted the translational potential of GWAS findings, i.e., to identify individuals at high risk of certain diseases, to inform precision medicine, for drug development, and to design gene therapy strategies [2, 120, 121]. With the cumulative cataloging and understanding of genetic modifiers of common diseases and advancement of genome editing technologies, therapeutics that strive to reproduce, mimic, or augment natural protective genetic variation should flourish in the future.
